# Beyond rehabilitation: toward developmental and trans-sectoral systems of support for complex acquired brain injury in children

**DOI:** 10.3389/fresc.2026.1936032

**Published:** 2026-10-01

**Authors:** Christiaan Gmelig Meyling, Sandra Bakker, Marike Reinders, Jeanine Voorman, Bas Oude Ophuis, Geeke Blenk, Hannemieke van der Lei, Marjolijn Ketelaar, Mireille Donkervoort, Jan Willem Gorter

**Affiliations:** 1 Research and knowledge hub, Netwerk Kind & NAH, Utrecht, Netherlands; 2 UMC Utrecht Brain Center and Center of Excellence for Rehabilitation Medicine, Utrecht University, Utrecht, Netherlands; 3 Parental Advisory Board, Netwerk Kind & NAH, Utrecht, Netherlands; 4 Department of Rehabilitation, Physiotherapy Science and Sports, UMC Utrecht, Utrecht, Netherlands; 5 Department of Pediatric Psychiatry, UMC Utrecht, Utrecht, Netherlands; 6 Department of Pediatric Oncology, Princess Máxima Center for Pediatric Oncology, Utrecht, Netherlands; 7 Regional ABI Expertise center, Amarant, Tilburg, Netherlands; 8 Child and Youth Center, Heliomare Rehabilitation, Heemskerk, Netherlands; 9 Research Centre of Social Innovations Flevoland, Windesheim University of Applied Sciences, Almere, Netherlands

**Keywords:** acquired brain injury, care network, children, chronicity, family, integrative care pathways, interdisciplinary care, rehabilitation

## Abstract

Pediatric acquired brain injury (ABI) can disrupt developmental trajectories across physical, cognitive, behavioral, emotional, and social domains. While rehabilitation pathways typically focus on recovery during the acute and subacute phases, many children and youth experience persistent or emerging difficulties long after formal rehabilitation treatment has ended. These difficulties may become more visible as developmental, educational, and social demands change, particularly during transitions across childhood and adolescence. Current systems, however, are often organized around sector-specific and time-limited models of service, resulting in fragmented support across healthcare, education, children and youth services, and settings. In this Perspective Paper, we introduce ‘pediatric ABI with complex consequences’ (ABI+) to draw specific attention to children and youth with ABI whose support needs in the community extend beyond acute care and sub-acute conventional rehabilitation pathways. ABI + is not proposed as a new diagnostic category, but as a vulnerable population as a result of ongoing dynamic interaction between neurological injury, child development, family functioning, and environmental factors over time. We argue that pediatric ABI rehabilitation should move from a phase-based, healthcare-centered model toward a developmental and trans-sectoral system of support. To illustrate this perspective, we describe the Dutch Pediatric ABI Network (Netwerk Kind & NAH), an integrated national network connecting community-based support, multidisciplinary expertise, research, policy, and lived-experience. Drawing on initial lessons learned from the development of this network, we aim to offer transferable insights that may inform the design of integrated systems supporting recovery, participation, and family functioning throughout the developmental course of children and youth with ABI.

## Introduction

Acquired brain injury (ABI) in children and youth refers to any non-congenital injury to the brain occurring after birth, including both traumatic brain injury (TBI) and non-traumatic brain injury (nTBI) etiologies such as stroke, brain tumors, encephalitis, and hypoxic injury (1). Pediatric ABI is a major cause of death and long-term disability among children and youth worldwide (2). The worldwide incidence of pediatric TBI ranges broadly and varies greatly by country, with most reporting a range between 47 and 280 per 100,000 children (3). In the Netherlands, extrapolation of hospital-based data suggests that approximately 19,000 children and youth aged 0–24 years receive a diagnosis of TBI or nTBI annually (4).

Children and youth with ABI are a heterogeneous group with respect to injury type, severity, age at injury, levels of impairments and consequences for participation in daily life situations (5). The sequelae of ABI may affect physical, cognitive, social, and emotional domains, often resulting in long-term restrictions in activities and participation at home, school, and in social life (6, 7). A defining characteristic of pediatric ABI is the disruption of ongoing developmental trajectories due to injury to the developing brain. As a result, difficulties may not only reflect the immediate effects of injury but may also emerge or become more noticeable as developmental, educational, and social demands increase. This contributes to unique long-term evolving trajectories post-ABI across childhood and adolescence into adulthood.

The management of pediatric ABI typically follows a trajectory across three interconnected phases (8). The acute phase focuses on diagnostics, medical stabilization, and prevention of secondary neurological damage (9). This is followed by the subacute rehabilitation phase, in which children with moderate to severe impairments receive multidisciplinary rehabilitation targeting recovery of motor, cognitive, and socio-emotional functioning (10, 11). In contrast, children with initially mild impairments are often discharged home without structured follow-up, placing them at risk for delayed identification of emerging problems later in life. In the chronic phase, the focus shifts toward optimizing participation in daily life (12).

Despite the recognized importance of discharge planning and community follow-up, many families experience persistent or emerging difficulties after formal rehabilitation treatment has ended (13–15). At this stage, the child, together with their family, is no longer a patient in a controlled clinical setting but an individual facing the full demands of daily life. As developmental and environmental demands change, previously subtle difficulties may become more apparent, with behavioral and cognitive difficulties often emerging as prominent and impactful manifestations. Parents, siblings, peers, teachers, and other involved caregivers may struggle to understand and respond to these challenges, particularly when support systems change or when the link between the struggles in everyday life and the earlier brain injury is no longer evident. Without adequate coordination and continuity of support, these evolving difficulties may contribute to cumulative complexity in functioning, including physical challenges, mental health problems, school failure, family stress, and reduced social participation (12, 16).

In this Perspective Paper we argue that the future of care for children and youth with ABI lies in a developmental, trans-sectoral approach. First, we characterize the vulnerable population ‘pediatric ABI with complex consequences’ (ABI+) to highlight the dynamic and evolving consequences of childhood-onset brain injury. Second, we discuss the key challenges that arise during transitions across care settings. Third, we describe the Dutch Pediatric ABI Network (“Netwerk Kind & NAH”) as an illustrative example of a layered ecosystem of expertise, connecting specialized healthcare, education, community services, research and family and lived-experience expertise. By doing so, we aim to contribute to care systems that support recovery, participation, and family functioning throughout the developmental course of children with ABI and their families.

## Characterizing pediatric ABI with complex consequences (ABI+)

Traditional approaches to classifying pediatric traumatic ABI primarily rely on indicators of acute injury severity, such as the Glasgow Coma Scale (GCS) and duration of post-traumatic amnesia (PTA) (17–19). While these indicators are useful for acute medical decision-making and early prognostication, they have limited value for understanding long-term functioning, participation and support needs (20). Children and youth with apparently mild brain injuries may develop persistent difficulties, whereas others with more severe injuries may show relatively favorable adaptation in supportive environments (21, 22). This variability illustrates that outcomes after pediatric ABI are not determined by injury characteristics alone, but by the dynamic interaction between neurological impairment, developmental processes, child factors and the family and environmental context (23–25).

To better capture this variability and developmental complexity, we introduce the term pediatric acquired brain injury with complex consequences (ABI+) to refer to a vulnerable population of children and youth with persistent or emerging difficulties across multiple domains after brain injury. ABI + is not proposed as a new diagnostic category, but as descriptive term to identify and characterize children and youth who experience persistent or emerging difficulties across multiple domains of functioning after brain injury and their families. As illustrated in Figure 1, these domains include physical and cognitive functions, daily functioning and participation, contextual factors, and risk and safety. By considering these domains in relation to one another, ABI + aims to support recognition of children and families whose evolving needs in the community are insufficiently addressed by rehabilitation pathways that primarily focus on acute and subacute recovery, rather than on long-term developmental support across the continuum of care and other services in the educational and social domain.

**Figure 1:**
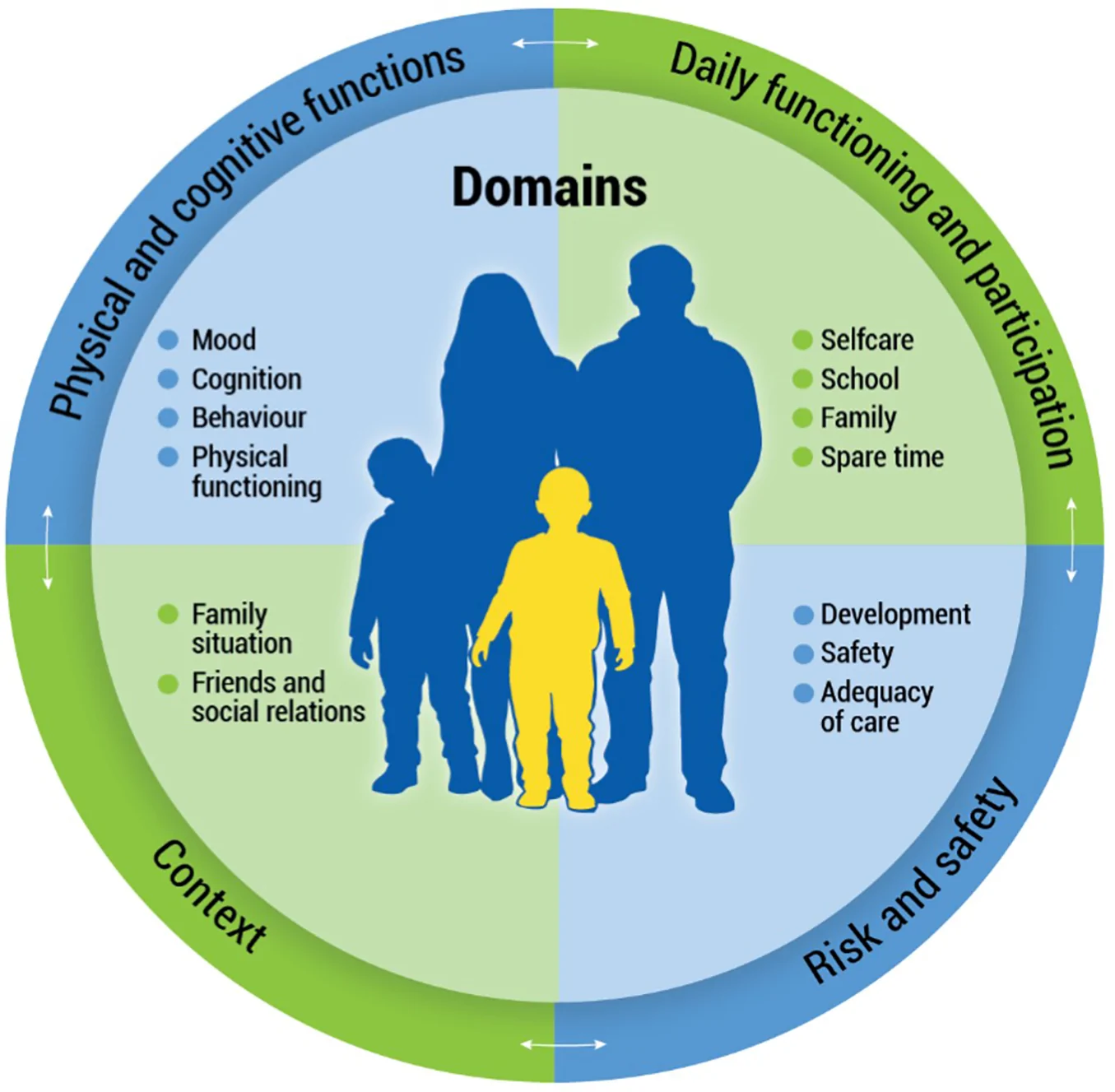
Domains of functioning to characterize children with ABI+.

A central feature of ABI + is the recognition that complexity after childhood brain injury emerges through the dynamic interaction between the child and the child's everyday contexts (21, 26). Neurobehavioral difficulties are often prominent long-term consequences and may resemble symptoms associated with a range of psychiatric conditions, including attention-deficit hyperactivity disorder, oppositional defiant disorder, anxiety, depression or autism spectrum disorder, complicating clinical interpretation and care allocation (27, 28). Yet complexity may also arise when the child's needs are insufficiently understood or accommodated within everyday contexts such as family and school. This is particularly relevant for children with mild/moderate or unrecognized brain injuries, or for those who did not receive formal rehabilitation treatment, as difficulties may only become apparent years later in school, family, or social contexts. As a result, children and their families often move between neurological, rehabilitation, and mental health care services without a shared understanding of the sequalae of ABI and language, contributing to fragmented and discontinuous care pathways.

From a developmental perspective, ABI + reflects the interaction between the timing of brain injury, the child's evolving developmental capacities, and the family and environmental context. As children grow older and face increasing cognitive, social, and educational demands, previously subtle impairments may become more apparent, a phenomenon often described as “growing into deficit” (29). These difficulties frequently often emerge during periods of transition, such as changing schools, entering adolescence, or changes in everyday support systems, such as a new teacher or sports coach. Consequently, trajectories following pediatric ABI are not static but continuously shaped by changing interactions between the child and their environment over time.

The approach in this Perspective paper aligns with established frameworks in health and development. The International Classification of Functioning, Disability and Health (ICF) conceptualizes disability as the result of interactions between health conditions, body functions and structures, activities, participation, and contextual factors (30). The ICF-framework has been operationalized in children through the six F-words – Functioning, Family, Fitness, Fun, Friends, and Future. The F-words have been increasingly integrated into paediatric care and research, and has been introduced to meaningfully and comprehensively address the relevant aspects of a child's life and their family (31, 32). Bronfenbrenner's ecological systems theory further emphasizes that child development unfolds through dynamic interactions within interconnected systems, including the family, school, and broader societal contexts (33). Together, these frameworks help explain why the consequences of pediatric ABI + evolve across developmental stages and life contexts, and why support needs frequently extend beyond the boundaries of traditional rehabilitation and healthcare systems.

The term pediatric ABI + has been recognized by the Dutch Government as a population at risk for long-term support needs in the community. Its added value lies in linking current support needs back to the history and timing of brain injury, while also emphasizing delayed recognition, changing developmental and environmental demands, and the need for coordinated long-term support across healthcare, education, youth services, and community settings.

## The gap between care and daily life

The mismatch between developmental trajectories and the organization of care becomes particularly visible during the transition from rehabilitation to daily life (34, 35). Although systems differ across countries, rehabilitation services, mental health care, educational systems, and social support are often organized in separate sectors with distinct professional frameworks and funding structures (36). As a result, collaboration across sectors may depend on individual professionals, rather than on embedded coordination structures (37). After formal rehabilitation treatment has ended, access to specialized ABI expertise may become limited, while responsibility for identifying emerging difficulties, providing support, and coordinating care shifts to schools, general practitioners, youth care providers, and community professionals (38). Moreover, the experience and knowledge gained during rehabilitation does not automatically translate into everyday environments such as school, sports, family life, and peer relationships. Professionals in these settings may struggle to link emerging behavioral or learning difficulties to a history of brain injury, particularly when the injury occurred years earlier (39). Consequently, families frequently become the de facto coordinators of care, navigating multiple systems in search of appropriate support on top of an already overwhelming situation after their child's injury (40). Within this fragmented landscape, professionals may each observe only part of the child's functioning, while no single professional or service has sufficient overview of the child's developmental trajectory, family context, and cross-domain support needs.

The relatively low prevalence and heterogeneity of pediatric ABI + further complicate the organization of care and research. Children with ABI + are dispersed across regions and settings, limiting opportunities for systematic data collection, knowledge accumulation, and development and implementation of care pathways (2). Bridging the gap between expertise and everyday environments requires new models of care that structurally connect healthcare, education, and community systems around children and their families (39).

## The Dutch pediatric ABI network as an illustrative example

To address the gaps in care for children and youth with ABI+, in the Netherlands the Dutch Pediatric ABI Network (Netwerk Kind & NAH) was established with a systemic, trans-sectoral approach. Rather than concentrating expertise within specialized institutions, the Network aims to develop and share expertise across the broader ecosystem surrounding children, youth and their families while maintaining strong connections between local practice, specialized assessment, and national knowledge development. In this model, expertise does not remain confined to specialized institutions, but travels through the network to reach schools, families, and community professionals (41).

We present the structure and operations of the Dutch Pediatric ABI Network as a practice-based example of how developmental and trans-sectoral support for children and youth with ABI may be organized. The Dutch Pediatric ABI Network operates through a layered model of expertise, combining community-based support, specialized multidisciplinary functional assessment, research and knowledge translation, and lived-experience involvement. (Figure 2). The impact and (costs)-efficiency of this national Network is yet to be evaluated.

**Figure 2:**
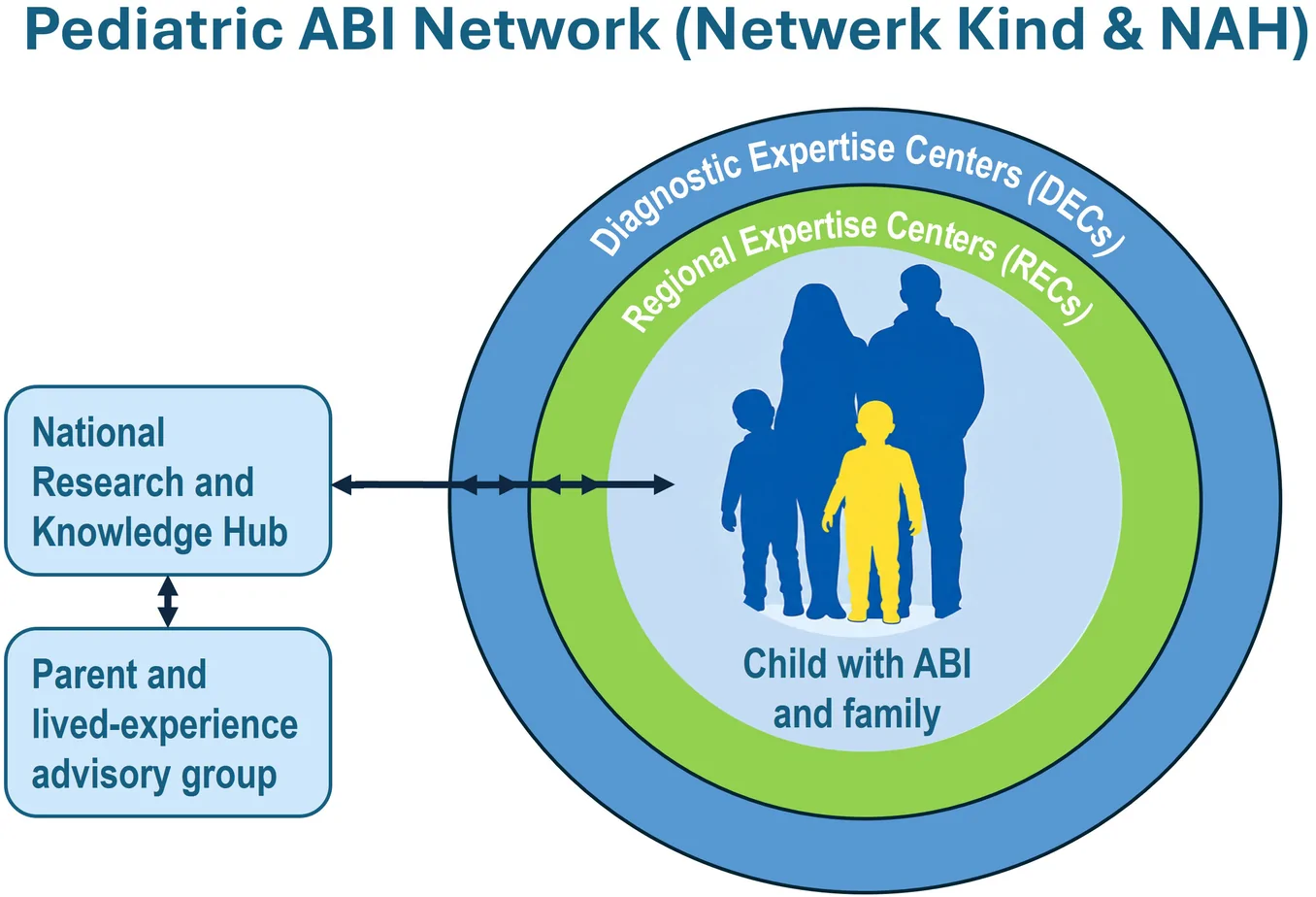
Layerd model of the Dutch Pediatric ABI Network.

### Layer 1: regional expertise centers

At the local level, six regional expertise centers (RECs) provide family-centered guidance, bridging the transition from clinical rehabilitation to everyday life. Case managers provide direct support to children and youth with ABI + and their families in their own environment. They conduct an initial inventory of the child's and family's concerns and help clarify the nature of emerging problems. Through their position within the network, case managers pediatric ABI know how to navigate the care landscape and connect families to appropriate expertise across sectors. In addition, they serve as accessible points of contact for schools and community services in coordinating care, translating specialized knowledge into practical strategies for everyday contexts, and identifying emerging developmental challenges at an early stage.

### Layer 2: diagnostic expertise centers

The Diagnostic Expertise Centers (DECs) provide a multi-specialist layer of expertise within the Network. The two DECs have a national coverage and integrate expertise from rehabilitation medicine, psychiatry, neurology, neuropsychology, and psychosocial disciplines. Children and youth with complex neurobehavioral or developmental challenges following ABI may be referred to a DEC for comprehensive multidisciplinary functional assessment and treatment advice. In addition, regional case managers and RECs can consult the DECs directly when they encounter persistent questions or need specialist advice. In this way, the DECs operate as consultation partners for the network ensuring consistency in the approach across levels.

### Research and knowledge hub & parent and lived-experience advisory group

At the national level, the Dutch Pediatric ABI Network functions as a research and knowledge hub. The hub brings together three complementary forms of knowledge: professional expertise, lived-experience knowledge, and research-based knowledge. Its role is to (a) support knowledge development, (b) gather questions and needs from practice and families, (c) translate knowledge into usable tools and guidance, and (d) disseminate insights across the network. In this way, innovations and insights from research and specialized services can be implemented in everyday care and educational settings, while challenges encountered in daily life directly inform research priorities, service development, and policy agendas.

The Network includes a structural parent and lived-experience advisory group. This advisory group ensures that children and youth with ABI and their families contribute to defining what constitutes good care, informing research priorities, and providing systematic feedback on the implementation of care pathways. Their involvement operationalizes the principle of “nothing about us without us” and strengthens the alignment between professional expertise and the realities of family life.

To date, the development of the Dutch Pediatric ABI Network has been supported by a combination of national funding and contributions from participating organizations. The Network has received financial and infrastructure support through the Dutch national program for Knowledge Networks for Specific Target Groups (“Kennisnetwerken voor Specifieke Doelgroepen”), funded by the Ministry of Health, Welfare and Sport and coordinated by ZonMw (2025–2026). In addition, start-up funding from the Dutch Healthcare Authority supported case managers for pediatric ABI.

This program aims to strengthen sustainable knowledge infrastructures for low-volume high-complexity populations through iterative evaluation, visitation, and implementation cycles. Continued funding is contingent upon ongoing development of expertise, collaboration, knowledge dissemination, and impact on care and support systems.

## Lessons learned from developing a trans-sectoral pediatric ABI network

The Dutch Pediatric ABI Network was initiated by professionals and organizations who recognized the need for more coordinated support for children and youth with ABI and their families. Initially, the Network developed largely through voluntary collaboration, professional commitment, and the energy of individuals willing to work beyond organizational and sectoral boundaries. This pioneering phase helped build relationships and a common understanding of the added value of a national trans-sectoral network. As the Network has grown, it has moved beyond this initial phase of formation toward a phase of consolidation, implementation, and growth. The development of the Dutch Pediatric ABI Network is an ongoing iterative process. Although the Network has not yet been formally evaluated as a model of care, its ongoing development has generated practice-based lessons that may be relevant for other (national) initiatives seeking to organize trans-sectoral support for children and youth with ABI and their families.

A first lesson is that trans-sectoral collaboration requires both commitment and structure. The development of the Network has been facilitated by the commitment and pioneering mindset of professionals, families, funding bodies and organizations willing to move beyond traditional sector boundaries. Regular national network meetings have supported knowledge exchange, alignment of practice, and collective problem-solving. These interactions have shown that professionals from different sectors often recognize the value of each other's expertise when opportunities for direct collaboration are created. At the same time, sustainable collaboration cannot rely solely on personal relationships or informal networks. As the Network matures, formalized care pathways, consultation structures and shared working procedures become increasingly important to ensure continuity and scalability.

A second lesson is that family and lived-experience involvement must be structurally embedded. Through the Network's advisory board, parents contribute to decision-making, identify emerging challenges, and help ensure that services remain aligned with the realities of daily life. Their involvement has consistently highlighted issues that may be overlooked within professional systems alone and has strengthened the relevance and legitimacy of network activities.

Despite these lessons, several challenges remain. First, sustainable funding continues to be a major constraint. The Pediatric ABI Network operates across multiple legislative and financial systems, including healthcare, education, social support, and youth services, each with its own priorities and funding mechanisms. As a result, investing in new cross-sectoral care structures is not always straightforward. Second, defining the ABI + population has proven challenging. No standardized approach exists to capture the multidimensional complexity of ABI + or to translate this complexity into criteria for identifying children who require specialized, long-term support. Third, nationwide coverage and equitable access remain works in progress. Although the Network continues to expand, access to specialized expertise and network services is not yet equally available across all regions. Strengthening regional capacity and ensuring that expertise reaches families, schools, and community professionals remain key priorities. Fourth, the development of sustainable data infrastructures remains a priority. The low prevalence and heterogeneity of pediatric ABI + make it difficult for individual institutions to generate robust evidence regarding long-term outcomes and service effectiveness (42, 43). Structured longitudinal data collection across the Network is therefore increasingly important, both to evaluate impact at the level of individual children and families as well as at the level of societal impact. It also allows for (health) system learning, quality improvement, and research at the population level. Developing such infrastructures requires agreement on shared outcomes, data governance, and long-term investment (44).

## Implications and future directions

The ideas presented in this Perspective Paper have implications for how systems for children and their families dealing with the long-term impact of ABI are organized. Rather than approaching pediatric ABI care as a linear pathway from acute treatment to rehabilitation and discharge, we propose a developmental and trans-sectoral model. Such a model recognizes that the consequences of ABI may evolve across childhood, adolescence and young adulthood, with support needs emerging during critical times of transition with changes in both the person and the environment. This includes the transition to adulthood, when young people are expected to take on new roles in further education, work, independent living, social relationships, and self-management of care.

The development of the Dutch Pediatric ABI Network aligns with broader developments in integrated care networks (ICN) across health systems. Integrated care models have been proposed as a response to fragmentation in service delivery, aiming to coordinate care across professionals, organizations, and sectors around the needs of specific population (45, 46). ICNs are increasingly implemented in both high-volume populations, such as dementia care, and low-volume, high-complexity conditions, including pediatric oncology and cerebral palsy (47–49). Across these contexts, integrated care approaches are closely aligned with contemporary policy frameworks such as the Quadruple Aim (49) and the Dutch Integrated Care Agreement (50), which emphasize improved outcomes, patient and family experience, professional well-being, and system sustainability through delivering the right care in the right place over time.

To determine whether a network model delivers its intended impact, future evaluation should include both process and outcome indicators. Process indicators may address case characteristics, regional coverage, waiting times, referral and consultation patterns, REC–DEC collaboration, use of shared working procedures, and participation in knowledge exchange activities. Outcome indicators may reflect what matters over time for children and families, including goal attainment, family centeredness of care, caregiver burden, child and youth well-being, sibling well-being, participation, school functioning, continuity of support, unmet support needs, and perceived added value by families and professionals. At the system level, evaluation should address whether the Network contributes to a more coherent care landscape and more appropriate use of specialized expertise across sectors. A key future direction is therefore the development of sustainable data infrastructures. The low prevalence and high heterogeneity of pediatric ABI + make it difficult for individual institutions to generate robust evidence on long-term outcomes and service impact. Knowledge development therefore requires data collection across regions, sectors, and levels of care. Structured longitudinal data collection within integrated networks can support individual follow-up, quality improvement, research, and policy development. This requires agreement on shared outcomes, data governance, privacy safeguards, and long-term investment. In this respect, pediatric ABI networks should increasingly function as learning systems in which clinical practice, research, implementation, policy, and lived-experience continuously inform each other (44, 51).

The Dutch Pediatric ABI Network has moved from an initial phase of formation, relationship-building, and role clarification toward a phase of consolidation, implementation, and growth. The current priority is to translate the network's shared vision into sustainable structures and care pathways, supported by longitudinal data infrastructure that enables learning and evaluation of societal impact. In doing so, the Network may help demonstrate how developmental and trans-sectoral rehabilitation systems can remain responsive to children's and families’ changing needs beyond formal treatment episodes.

## Data Availability

The original contributions presented in the study are included in the article/Supplementary Material, further inquiries can be directed to the corresponding author.
